# Wave directional spreading from point field measurements

**DOI:** 10.1098/rspa.2016.0781

**Published:** 2017-04-26

**Authors:** M. L. McAllister, V. Venugopal, A. G. L. Borthwick

**Affiliations:** Institute for Energy Systems, University of Edinburgh, Kings Buildings, Edinburgh EH9 3JG, UK

**Keywords:** surface gravity waves, directional spreading estimation, point measurement, second-order difference, *in situ* wave data

## Abstract

Ocean waves have multidirectional components. Most wave measurements are taken at a single point, and so fail to capture information about the relative directions of the wave components directly. Conventional means of directional estimation require a minimum of three concurrent time series of measurements at different spatial locations in order to derive information on local directional wave spreading. Here, the relationship between wave nonlinearity and directionality is utilized to estimate local spreading without the need for multiple concurrent measurements, following Adcock & Taylor (Adcock & Taylor 2009 *Proc. R. Soc. A* **465**, 3361–3381. (doi:10.1098/rspa.2009.0031)), with the assumption that directional spreading is frequency independent. The method is applied to measurements recorded at the North Alwyn platform in the northern North Sea, and the results compared against estimates of wave spreading by conventional measurement methods and hindcast data. Records containing freak waves were excluded. It is found that the method provides accurate estimates of wave spreading over a range of conditions experienced at North Alwyn, despite the noisy chaotic signals that characterize such ocean wave data. The results provide further confirmation that Adcock and Taylor's method is applicable to metocean data and has considerable future promise as a technique to recover estimates of wave spreading from single point wave measurement devices.

## 1. Introduction

The relative directions in which wave components travel have a substantial effect on their combined kinematics and dynamics. This has significant implications in coastal and ocean engineering, including design codes for offshore oil platforms, ship safety, optimization of wave energy devices and the dispersion of oil slicks and plastic contaminants. For the majority of wave observations made in the oceans, the relative directions of the underlying wave components remain unknown because the observations are usually made in isolation as point measurements. The conventional way of overcoming this problem is to use either an array of three or more instruments [1,2] or multi-degree-of-freedom devices [3–5] such as radar, clover-leaf buoys and wave-rider buoys.

Another approach is to exploit the relationship between wave nonlinearity and wave directionality. The majority of waves in the ocean are weakly nonlinear; therefore, their observed time series will contain second-order nonlinearities [6,7]. The connection that exists between nonlinearity and directionality as observed experimentally by Johannessen & Swan [8], Onorato *et al.* [9], and in the field by Forristall [10], and Toffoli *et al.* [11], provides a means by which information on local directional spreading can be inferred from the nonlinearites that lie within a single point measurement. To exploit this connection, it is necessary to extract the linear free waves and second-order bound waves from the wave elevation time signal, and then fit second-order wave theory to the bound wave signal using an assumed spreading function, and hence estimate the spreading from the optimal fit. Walker *et al.* [12] presented an approximate method for separating out the linear free waves and second-order bound waves contained within an observed free surface elevation time series, through filtering in the frequency domain and minimizing skewness. For a prescribed wave spreading function involving a combination of interacting freely propagating linear waves, the resulting bound waves can be calculated to second-order using interaction kernels derived for finite depth by Dalzell [13]. Using this theory, the bound waves associated with a given set of free waves are simply a function of their component directions. By comparing the predicted bound second-order difference waves for an assumed spreading distribution to the actual bound second-order difference waves contained within the original measurement, Adcock & Taylor [14] derived a method for estimating the local directional spreading for a point measurement. This approach, herein called the ‘long-wave method’ (LWM), was satisfactorily validated using experimental data obtained by Cornett *et al.* [15] and numerically generated data with the addition of random noise. Adcock *et al.* [16] used the same approach to infer the conditions that gave rise to the Draupner wave of 1 January 1995 from information acquired by a single point gauge, and found that directional spreading was of key importance in understanding the extreme event. The results were found to be consistent with other nearby observations [17,18].

Here, Adcock and Taylor's method is applied to a large dataset of observations from three measurement devices located on the North Alwyn platform in the northern North Sea. By examining high-quality data signals from all three devices, comparison is made between estimates of directional spreading using the single probe LWM and conventional methods. Where data are of insufficient quality from any of the three devices, comparison is drawn with hindcast data obtained from the European Centre for Medium-Range Weather Forecasts (ECMWF).

The paper is structured as follows. Section 2 describes the data sources. Section 3 presents the methodology. Section 4 investigates the sensitivity of the method. Section 5 details the results. Section 6 lists the conclusions.

## 2. Data

### (a) North Alwyn platform

The ocean wave data considered herein were all obtained from single point gauges located on the North Alwyn platform, a fixed jacket offshore structure located in the northern North Sea at (60°48.5′ N, 1°44.17′ E) about 150 km east of the Shetland Isles. Mean water depth at the platform was 130 m. The platform comprised two structures, North Alwyn A and North Alwyn B, connected by a bridge. The platform substructure was sparse, with each support column of diameter *D*=1.5 m. The wavelength of incoming waves was typically of the order of *L*=100 m. Hence, *D*/*L*≪0.2 and the North Alwyn platform can be considered as being composed of small diameter cylinders, meaning that wave–structure interactions such as reflections and diffraction were negligible [19] and did not significantly affect the recorded observations of the surrounding wave field.

**Figure 1.**
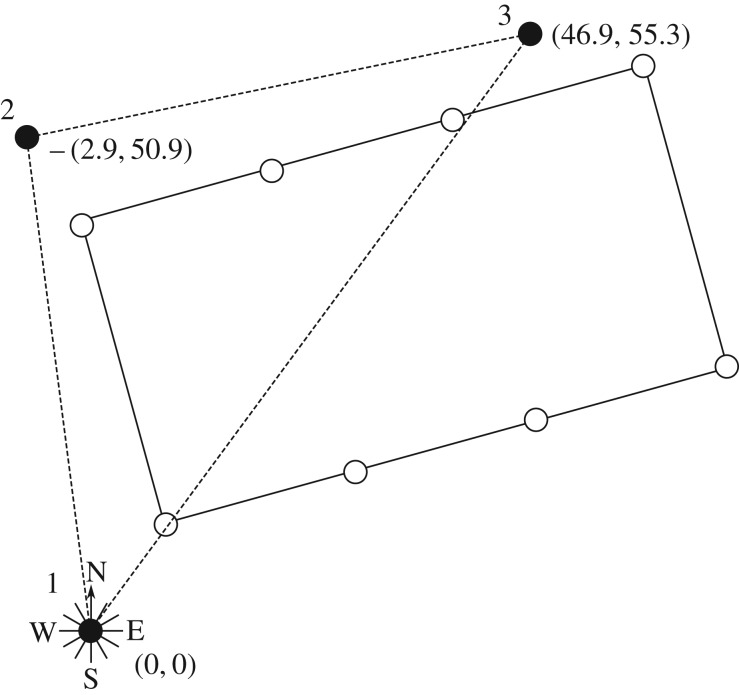
Schematic showing arrangement of North Alwyn measurement array in plan.

The measurement devices were all located on North Alwyn A, and comprised three Thorn EMI infrared laser probes that simultaneously measured sea surface elevations (figure 1). The probes were set out in plan as nodes of a triangle with side lengths of about 50, 51 and 72.5 m. The probe resolution was ±5 cm (i.e. accurate to within 1%). It should be noted that the water elevation time series measured by the probes contained a number of wild points and occasional signal drop-out exhibited as plateaux in the data [20]. A Labtech Notebook on the platform was used to control data acquisition, undertake preliminary data processing, and provide local data storage. The PC acquired data at 5 Hz, via an XE software package. Raw data were split into 20 min blocks for statistical treatment. Further details are given by Wolfram *et al.* [21].

Free surface elevation data were recorded over a 10 year period from 1994 to 2004. The data measurements were continuously monitored, and values stored when either the significant wave height *H*_s_ exceeded 3.5 m or the wind speed was greater than 16 ms^−1^. A total of 448 individual ‘storms’ made up the North Alwyn dataset, comprising 16 422 separate 20 min duration files, each containing three concurrent sets of measurements. Prior to analysis, data were screened using a strict quality control process based on a procedure outlined by Christou & Ewans [20,22]. All data that passed these tests were subjected to further analysis. Directional analysis was performed by conventional methods when the measurements from all three probes were simultaneously of good quality. Table 1 summarizes these results, listing the number of measurements that passed the quality control process. Of the unprocessed measurements, 20 802 from any of the three sensors passed the quality control procedure, and of these 391 were suitable for multi-probe directional analysis.

**Table 1.** North Alwyn Dataset: availability of measurements between 1994 and 2005, and results of quality control process.

year	storms	files	QC passed	3 probes QC passed
1994	1	177	172	124
1995	5	658	595	0
1996	1	71	148	22
1997	5	1241	1659	0
2000	176	5972	7698	185
2001	100	2272	2581	12
2002	108	4019	4792	44
2003	52	2012	3157	4
total	448	16 422	20 802/49 266	391/16 422

### (b) Hincast data from European Centre for Medium Range Weather Forecasts

The ECMWF provides publicly available meteorological datasets produced by hindcast models. The data used herein were taken from the ERA-20C model, which provided hindcast atmospheric information based on an assimilation of historic global observations for the entire twentieth century. Model outputs included a wide range of meteorological data; of these, the key metocean parameters were hindcast using a WAM model. The WAM model outputs spectra, discretized at 36 directions and frequencies. The ‘wave spectral directional width’ *σ*_E_ output had a spatial resolution of *δx*=28 km and temporal resolution of *δt*=3 h. A full specification of the wave model is given by ECMWF [23].

## 3. Method

### (a) Linearization

In order to calculate the bound waves for a given time series of free surface elevation above the mean water level *η*(*t*), the linear free waves *η*_L_ must first be extracted. The data were linearized following the procedure outlined by Walker *et al.* [12]. Second-order difference waves *η*_2F−_ were removed by high-pass filtering the data at half the peak frequency. Second-order sum nonlinearity was then removed by adjusting the Stokes’ *S*_22_ parameter to minimize the skewness of the resulting linear time series.

3.1 $$\eta_{L}=\eta_{F}-\frac{S_{22}}{h}(\eta^{2}-{\hat{\eta }}^{2}),$$

where *h* is the mean water depth, and *η*_L_ is the linearized, *η*_F_ is the high-pass filtered, and $\hat{\eta }$ is the Hilbert-transformed, time series obtained from *η*. The skewness of *η*_L_ is calculated using

3.2 $$\gamma =\frac{1}{N\sigma^{3}}\sum_{n=1}^{N}\eta_{L}^{3},$$

where *N* is the number of data points and *σ* is the standard deviation of the linearized time series.

### (b) Second-order calculations

Wave–wave interaction of linear freely propagating waves causes nonlinear bound waves which occur at increasing order with decreasing amplitude. Second-order bound waves occur as a result of interacting wave pairs, at the sum and difference of their constituents. The sum terms occur in the tail of the spectrum and overlap the linear waves. However, the difference terms occur at the low end of the spectrum where they dominate linear waves, owing to the faster decay of the linear spectrum. This makes it easier to separate out the difference waves.

Difference waves resulting from a linear spectrum may be calculated using the interaction kernels derived by Dalzell [13] for finite depth. Starting with the linear spectrum expressed as a complex Fourier series

3.3 $$\eta =\sum_{n=1}^{n=N}x_{n}\exp(i\omega_{n}t),$$

in which *x*_*n*_ is the *n*th constituent of the complex vector **x** generated by performing a fast Fourier transform (FFT) on the free surface time series, *ω*_*n*_ is the corresponding angular frequency of the *n*th constituent, and *t* is time. Calculating the interaction of each wave pair gives the second-order difference waves

3.4 $$\eta_{2-}=\sum_{i=1}^{i=N}\sum_{j=1}^{j=N}\sum_{n=1}^{n=N}\sum_{m=1}^{m=N}d(\theta_{i})x_{n}\;d(\theta_{j})x_{m}K^{-}\exp(i(\omega_{n}-\omega_{m})t),$$

where *d* is the assumed spreading function, dependent on wave incidence angle *θ*, and *K*^−^ is the interaction kernel for difference terms. *K*^−^ is a function of *ω*, *θ*, *h*, and wavenumber *k*, and is defined as

3.5 $$\begin{array}{rl} K^{-} & \;=\frac{\omega_{n}^{2}+\omega_{m}^{2}}{2g}+\frac{\omega_{n}\omega_{m}}{2g}\left(1+\frac{\cos(\theta_{n}-\theta_{m})}{\tanh(|k_{n}|h)\tanh(|k_{m}|h)}\right)\\ & \;\;\times \left(\frac{{\left(\omega_{n}-\omega_{m}\right)}^{2}+g|k_{n}-k_{m}|\tanh(|k_{n}-k_{m}|h)}{D_{m}(k_{n},k_{m})}\right)\\ & \;\;+\frac{\left(\omega_{n}-\omega_{m}\right)}{2gD_{m}(k_{n},k_{m})}\left[\frac{\omega_{n}^{3}}{\sinh^{2}(|k_{n}|h)}-\frac{\omega_{m}^{3}}{\sinh^{2}(|k_{m}|h)}\right] \end{array}$$

where

3.6 $$D_{m}(k_{n},k_{m})={\left(\omega_{n}-\omega_{m}\right)}^{2}-g|k_{n}-k_{m}|\tanh(|k_{n}-k_{m}|h).$$

#### (i) Numerical implementation

Equation (3.4) involves a quadruple summation over both frequency and direction. This incurs large computational cost, which is reduced using the following procedure. Dropping the exponential term in equation (3.4), the complex vector that represents the difference waves may be expressed as

3.7 $${\text{x}}_{2-}=\sum_{i=1}^{i=N}\sum_{j=1}^{j=N}\sum_{n=1}^{n=N}\sum_{m=1}^{m=N}d(\theta_{i})x_{n}\;d(\theta_{j})x_{m}K^{-}.$$

Noting that d(*θ*) is the only independent variable in equation (3.7) for a given time series, then *K*^−^ can be expressed as an element of the *n*×*m* matrix **K**^**−**^(*θ*_*δ*_), where *θ*_*δ*_ is simply the angle between interacting wave pairs. Hence, for frequency-independent cases *x*_*n*_*x*_*m*_ and d(*θ*_*i*_) d(*θ*_*j*_) can be expressed in matrix form as

3.8 $$X(n,m)={\text{x}}^{T}\text{x},$$

and

3.9 $$D(i,j)={\text{d}}^{T}\text{d}.$$

***D*** can be transformed to a function of *θ*_*δ*_=*θ*_*i*_−*θ*_*j*_, by summation over its diagonals

3.10 $$\text{d}(\theta_{\delta })=\sum_{i-j=0}^{i-j=N}D_{ij}.$$

Summing over all angles of separation gives the complex matrix ***X***_**2−**_, which represents the interaction of all frequency pairs over all directional pairs. Diagonal elements of this matrix represent values of equal resultant frequency, therefore summation over diagonals provides the complex vector **x**_**2−**_ from

3.11 $${\text{x}}_{\text{2}-}=\sum_{n-m=0}^{n-m=N}X_{2{-}_{nm}},$$

where

3.12 $$X_{\text{2}-}(n,m)=\sum_{\delta =0}^{\delta =N}d(\theta_{\delta })XK^{-}(\theta_{\delta }).$$

Finally, performing the inverse FFT on **x**_**2−**_ gives the second-order difference waves

3.13 $$\eta_{2-}=\sum_{n=1}^{n=N}x_{2{-}_{n}}\exp(i\omega_{2{-}_{n}}t),$$

in which *ω*_2−,*n*_ is the *n*th difference frequency.

**Figure 2.**
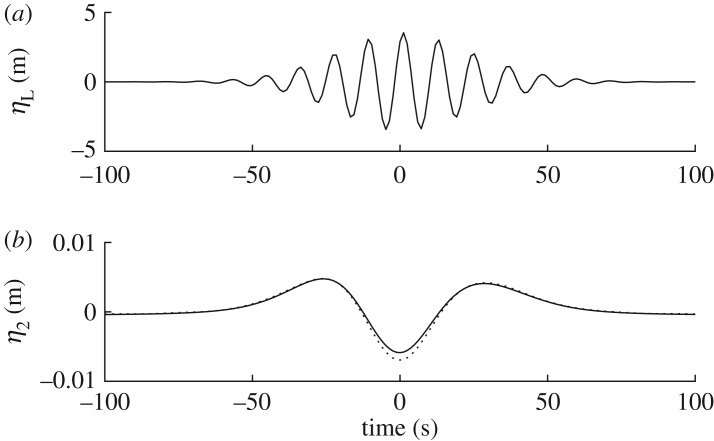
NewWave verification: (*a*) linear focused wave group free surface elevation time series *η* at *x*=0; and (*b*) second-order difference wave time series showing interaction kernels *η*_2*T*−_ (solid line) and fully nonlinear potential flow *η*_2F−_ (dashed line) [24].

This method was tested against fully nonlinear potential flow simulations of a directionally spread focused NewWave group on water of infinite depth carried out by Gibbs & Taylor [24], using a numerical solver developed by Bateman *et al.* [25]. Figure 2 shows the linear free surface of the simulations and the corresponding bound difference waves obtained using the present method and that of Gibbs & Taylor [24]. Excellent agreement is obtained regarding the long waves; there is nevertheless a slight discrepancy at focus which is the result of narrowing of the directional spectrum due to nonlinearity.

### (c) Spreading estimation

#### (i) Point measurement: long-wave method

Theoretical difference waves can be produced for any arbitrary spreading distribution using equation (3.5) to (3.13) for the linearized time series *η*_L_. Here, the predicted difference waves, *η*_2_T−__, are compared with observed difference waves, *η*_2_F−__ from (3.13), that were extracted by filtering the measured free surface time series *η*. By taking the Euclidean norm,

3.14 $$\Delta_{\mathrm{LW}}=\frac{\sqrt{\sum {\left(\eta_{2_{T-}}-\eta_{2_{F-}}\right)}^{2}}}{\sqrt{\sum {\left(\eta_{2_{F-}}\right)}^{2}}}.$$

The value of Δ_LW_ is then minimized by varying the assumed spreading distribution. In this case, a wrapped-normal distribution is used, where the spreading value *σ* corresponds to standard deviation of a normal distribution wrapped around a full circle

3.15 $$d(\theta )=\frac{1}{\sigma \sqrt{2\pi }}\exp\left(-\frac{\theta^{2}}{2\sigma^{2}}\right).$$

It is assumed that spreading is frequency independent. The value *σ*_opt_ that minimizes Δ_LW_ and hence provides the optimum fit between the observed and theoretical difference waves *η*_2_T−__ and *η*_2_F−__ is determined as the best estimate of the local spreading.

By performing the foregoing LWM analysis on the entire dataset, any data for which the method did not successfully minimize the long-wave difference between 0–90° were discarded. Data where freak waves were located were also removed, because such waves have been shown to cause anomalous second-order difference waves [16]. Here, a freak wave is defined as a wave with up or down-crossing height greater than twice the significant wave height *H*_*m*0_ [26].

#### (ii) Array measurements

Where data of sufficiently high quality were simultaneously available from the three gauges at North Alwyn, the iterative maximum likelihood method (IMLM) is used to estimate the directional spectrum d(*θ*)*s*(*ω*). From this spectrum, the spectrally weighted frequency-independent spreading distribution d(*θ*) is calculated, and a wrapped-normal spreading distribution with standard deviation *σ*_*D*_ fitted to d(*θ*).

## 4. Sensitivity

### (a) Goodness of fit

**Figure 3.**
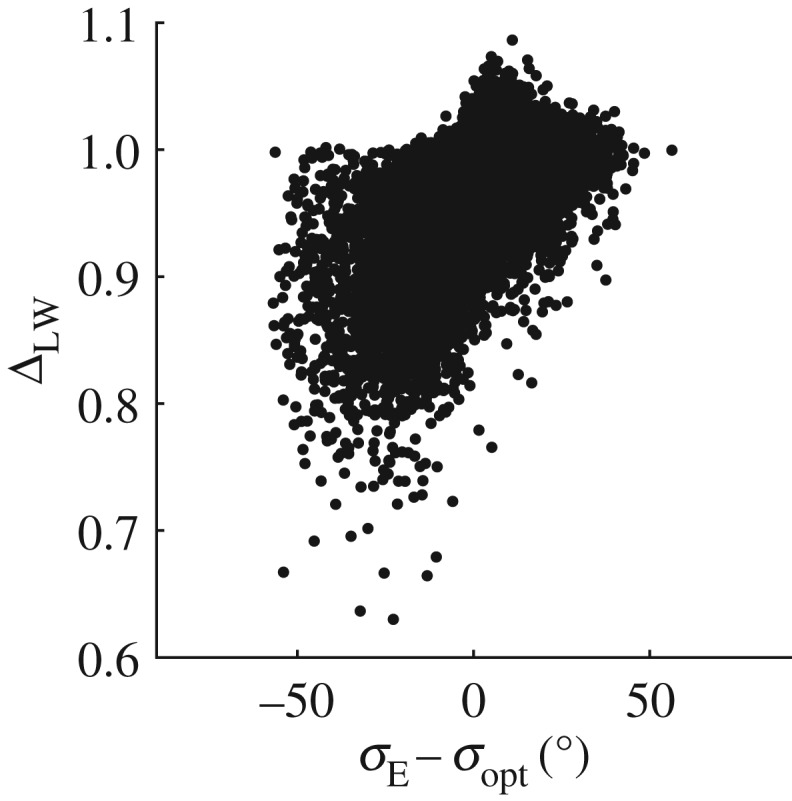
Scatter plot of goodness-of-fit estimates against spreading error for the LWM compared with the ECMWF hindcast.

**Figure 4.**
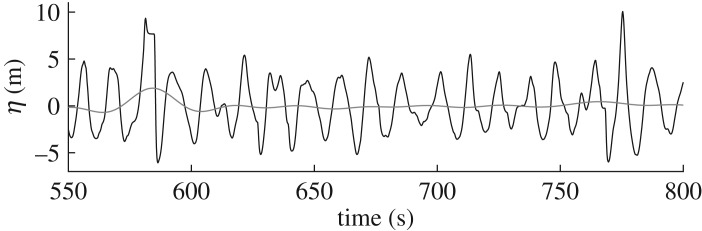
Free surface elevation and second-order difference time series at North Alwyn for a selected storm where there is a large set-up in second-order waves owing to measurement error: *η*(*t*) free surface elevation (black); and *η*_2F−_(*t*) filtered difference waves (grey).

**Figure 5.**
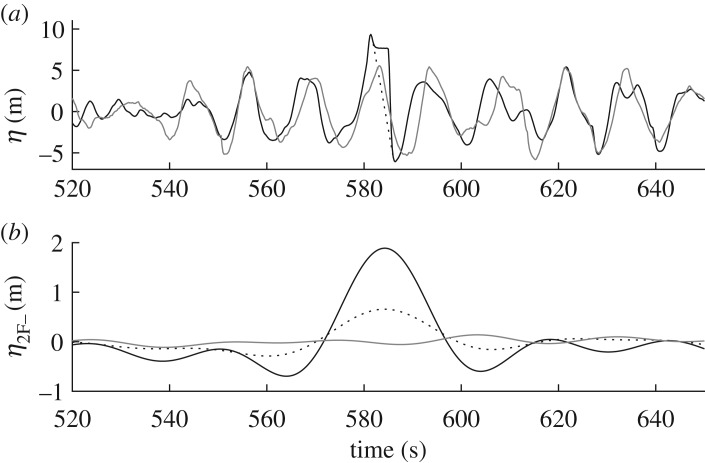
Wave free surface elevation time series at North Alwyn showing unmodified signal (Probe 1, solid black line), smoothed signal (Probe 1, black dashed line) and concurrent measurement from Probe 3 (grey line): (*a*) free surface elevation time series, *η*(*t*); and (*b*) filtered second-order difference waves *η*_2F−_(*t*).

The long-wave difference Δ_LW_ is primarily used as a goodness-of-fit parameter, in finding the optimal assumed spreading value *σ*_opt_. However, Δ_LW_ also provides information on the quality of fit that was achieved in finding *σ*_opt_. High values of Δ_LW_ corresponding to *σ*_opt_ imply that the fit may not be particularly satisfactory despite *σ*_opt_ being the best fit. Where a good fit is not achieved, the accuracy of *σ*_opt_ may be affected adversely. Figure 3 shows how Δ_LW_ varies against the spreading error *σ*_E_−*σ*_opt_, where *σ*_E_ is the value of spreading predicted by the ECMWF hindcast. In this instance, hindcast data are used for mainly illustrative purposes; the validity of this comparison is discussed in §5a. However suitable the comparison may be, large differences in spreading are clearly indicative of error. For $\Delta_{\mathrm{LW}}⪆1$, the error *σ*_opt_−*σ*_E_→+, meaning *σ*_opt_<*σ*_E_ and the spreading is underestimated. As Δ_LW_→0, the error also reduces. However, for Δ_LW_<0.8, the spreading is overestimated. In such cases a seemingly good fit is achieved (as indicated by the very low value of Δ_LW_) despite a large discrepancy in the predicted value of spreading. Visual inspection of the results indicated that this arose from erroneous measurements. Figure 4 shows an example of this type of error, where near flat sections or sudden changes in slope that are missed by the quality control process cause pronounced set-ups in the filtered second order difference waves *η*_2F−_. In this example, there are two similarly sized waves in *η*(*t*) occurring at around 580 and 775 s, of crest height ∼10 m. Both waves cause set-ups in *η*_2F−_ as may be expected for large amplitude crests. The set-ups associated with the waves are of amplitude ∼2 m and ∼0.5 m, respectively. The flat section measured in the first wave is clearly an error, and not an artefact of the wave profile. This type of error typically occurs when the sensor loses the free surface temporarily and logs the previously recorded value until the surface is found once more. To assess the effect this error has on *η*_2F−_, the flat error was artificially smoothed out as shown by the dashed line in figure 5*a*. Referring to the array shown in figure 1, the solid line corresponds to measurements at Probe 1 and the grey line to Probe 3 for comparison. Measurements at Probe 2 are omitted in figure 5 owing to poor quality.

Linear interpolation was used to remove instrumentation error at the crest of the first large wave, and so the resulting shape of the wave measured at Probe 1 is slightly larger but not dissimilar in shape to the corresponding wave recorded at Probe 3 figure 5*a*. Filtering the modified time series to obtain *η*_2F−_ results in the dashed line shown in figure 5*b*; the black solid line shows *η*_2F−_ for the unmodified time series. The modified time series has an amplitude of ∼0.65 m, which is comparable to that associated with the second large wave at 775 s.

The LWM spreading estimates from the modified and unmodified time series are 34.6° and 86.3°, respectively. The modified value concurs with the prediction made by the ECMWF hindcast of 30.8°, as well as the other values of *σ*_opt_ estimated during the rest of the storm.

This approach was extended to Storm 369 of the North Alwyn dataset, discussed in more detail in §5b. This storm was selected because it possesses three outlying results that derive from obvious measurement errors that have passed the QC process. Additionally, 15 measurements taken during the storm allow for IMLM estimation of *σ*_D_, which provides further confidence in the expected values of spreading. Again, available concurrent measurements were used as a guide when smoothing out measurement errors. Table 2 lists the results obtained after modifying the erroneous time series. The modified estimates of *σ*_opt_ lie much closer to the values of both *σ*_E_ and *σ*_*D*_ which indicate that the *σ*≈31° for Storm 369. For all three cases, this relatively simple approach reduces the associated error in predicted spreading, despite reducing the goodness of fit. The interpolation method used to smooth out errors has a significant effect on the estimate of *σ*_opt_. As there is no way of establishing for certain the true waveform, it is not suggested that this approach is used as a means of producing spreading estimates. However, the results presented here illustrate a particular source of sensitivity which does not lie with the LWM itself. It is obvious that errors undetected by the quality control process can result in gross overestimation of the spreading. Herein, visual inspection was used. In practice, a more robust means of error detection is desirable.

**Table 2.** Spreading estimates produced using raw and modified time series which correspond to three outlying estimates of *σ*_opt_ in Storm 369, that were a result of measurement errors.

	raw		modified	
outlier	*σ*_opt_(°)	Δ_LW_	*σ*_opt_(°)	Δ_LW_
(*a*)	76.6	0.942	39.6	0.971
(*b*)	46.4	0.915	31.1	0.989
(*c*)	52.4	0.839	28.1	0.996

#### (i) NewWave comparison

NewWave [27], depicted in figure 2*a*, constitutes a focused wave group where all free wave components are in phase at *x*=0 and *t*=0. In practice, this waveform is used as a design wave because it provides an accurate approximation to the shape of the largest waves contained within ocean observations (e.g. [28]). Similarly, the second-order difference waves *η*_2NW−_ corresponding to this profile can be used to provide an approximation to those expected in large wave events [29]. The phasing and shape of actual waves affect the amplitude of *η*_2F−_, causing some scatter about the NewWave approximation. Even so, this approach provides a general guide to the amplitude that should be expected for a given set of conditions. For a given spectral density *S*(*ω*), the corresponding new wave profile is calculated as

**Figure 6.**
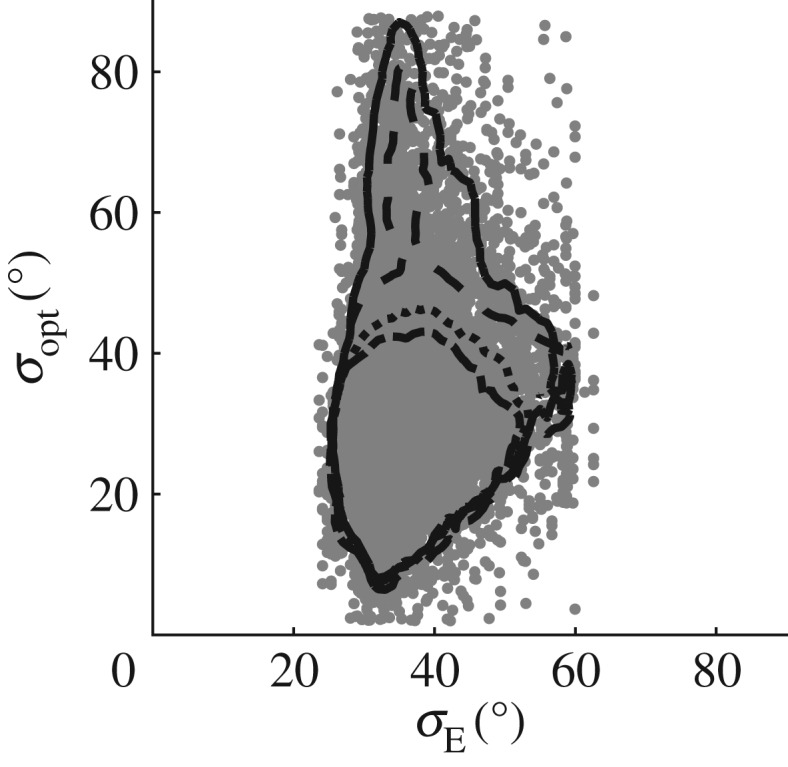
Scatter diagram showing LWM estimate spreading values filtered using NewWave amplitude ratio, *a*_−2_/*a*_−2NW_ plotted against hindcast estimates of spreading (grey dots), with superimposed contours showing 95% confidence values; results screened using amplitude ratio *a*_2−_/*a*_2NW−_ values less than 100 (solid line), 25 (dashed line), less than 10 (dotted line) and less than 5 (dot-dashed line).

4.1 $$\eta_{\mathrm{NW}}(t)=\frac{\sum_{n=1}^{N}S(\omega_{n})\cos(\omega_{n}t)}{\sum_{n=1}^{N}S(\omega_{n})},$$

in which *t* is time from focus, located at *x*=0. Using the linear NewWave profile *η*_NW_, the second-order difference bound waves *η*_2NW−_ are calculated using equation (3.4). The second-order difference amplitude associated with the NewWave profile normalized by significant wave height *a*_2NW−_/*H*_s_ can then be used to establish whether or not the observed set-up in amplitude *a*_2F−_/*H*_s_ found in *η*_2F−_ is feasible. When normalized in this way, the value of *a*_2NW−_/*H*_s_ depends upon the peak period *T*_*p*_ and spreading angle, with the assumption that the corresponding linear amplitude of the NewWave profile is *a*/*H*_s_=1 for a JONSWAP spectrum with peak enhancement factor 3.3. Therefore, a NewWave amplitude can be calculated for each measurement using the appropriate values of *T*_*p*_ and *σ*_E_. The ratio of *a*_2F−_/*H*_s_ to *a*_2NW−_/*H*_s_ provides an understanding of whether the set-up in *η*_2F−_ is likely to be the result of a large wave or arising from an anomaly. Figure 6 shows the effect of using *a*_2F−_/*a*_2NW−_ as a quality control parameter. Values of *σ*_opt_ are plotted against the corresponding values of *σ*_E_. Overlaid are the 95% contours when the data are screened using decreasing ratios of *a*_2F−_/*a*_2NW−_ from 100 to 5. At high ratio of *a*_2F−_/*a*_2NW−_ the amplitude observed in the measurement is much larger than would reasonably be expected; conversely as the ratio is reduced, results where the long wave estimate is grossly overestimated are progressively filtered out. This further illustrates the method's sensitivity to measurement errors. The results from the North Alwyn data confirm that NewWave provides an effective *a posteriori* method for error detection.

### (b) Cut-off frequency

Abnormal set-ups in the filtered difference waves, *η*_2F−_, have been shown above to result from errors in the measured time series *η*. Moreover, if the linear spectrum does not decay as sharply as expected, large-amplitude linear components arise in *η*_2F−_ that can dominate the smaller-amplitude second-order components. The resulting increases in *η*_2F−_ amplitude may adversely affect the values estimated by the LWM. To overcome this, we reduce the frequency at which the original data *η* are filtered in order to obtain *η*_2F−_ which is uncontaminated by the effect of any linear components that lie close to the original frequency cut-off. This was examined by applying the LWM to the entire dataset with difference waves filtered at 0.5*f*_p_, 0.4*f*_p_ and 0.3*f*_p_. No difference in the estimated values of *σ*_opt_ was discernible when different frequency cut-offs were used.

## 5. Results

### (a) Correlation with European Centre for Medium-Range Weather Forecasts hindcast

For the majority of the data, measurement quality does not permit conventional directional estimation, and so an indirect means of comparison is necessary. Here, the ‘wave spectral directional width’ *σ*_E_ produced by the ECMWF ERA-20C model is used. The indirect nature and low resolution relative to that of the North Alwyn dataset means that direct comparison between the values of *σ*_opt_ and *σ*_E_ is of limited use on a measurement by measurement basis. However, as opposed to comparing individual values, examination as to how the two sets of data correlate in time is much more informative. From figure 6, it is apparent that the values of spreading produced by the hindcast model are limited to the range of approximately 20°≤*σ*_E_≤60°, this may be a result of the 10° directional resolution of the hindcast model. It is therefore not possible to assess values of *σ*_opt_<20°, through this means of comparison.

**Figure 7.**
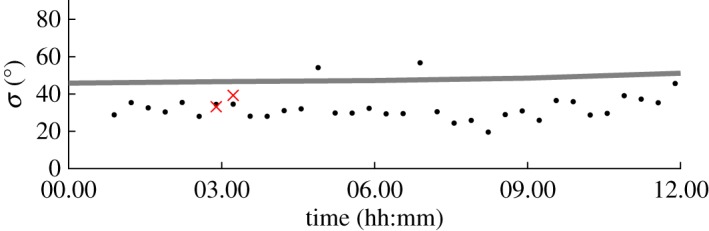
Storm 50 of the North Alwyn dataset which shows a bias between ECMWF and LWM estimates of directional spreading: *σ*_opt_ (black dots); *σ*_E_ (grey line); and *σ*_*D*_ (×). (Online version in colour.)

The majority of storms in the North Alwyn dataset are relatively short in duration with an average length of 12 h, and consequently their spreading shows little temporal variation. For such storms, it is difficult to find correlation, and accordingly it is difficult to establish whether either *σ*_opt_ or *σ*_E_ correctly predict spreading if their values are different. Figure 7 shows an example of a storm where this is the case. Both the values of *σ*_opt_ and *σ*_E_ show little variation over the duration of the storm, with a gradual increase in spreading as the storm progresses and a mean difference of ∼15°, and it is difficult to draw any immediate conclusions. However, for this particular storm two of the measurement files were suitable for conventional estimation, as shown by the crosses. At about 03.00, the hindcast prediction *σ*_E_=46.6° and the mean of two values either side of 03.00 are 36.2° and 34.5° for *σ*_*D*_ and *σ*_opt_, respectively. This limited additional information suggests that the values of *σ*_opt_ are more likely to be correct, and the hindcast model overestimates the spreading.

When there is variation in spreading predicted by the hindcast model it is possible to establish whether *σ*_opt_ is correctly measuring the local spreading. If a strong correlation exists between the values of *σ*_opt_ and *σ*_E_, it is clear that the hindcast is modelling the same conditions as the LWM is measuring. A means of parametrizing the correlation observed between the two variables is the correlation coefficient. The covariance of two variables *A* and *B* is given by

5.1 $$\mathrm{cov}(A,B)=\frac{1}{N-1}\sum_{i=1}^{N}(A_{i}-\mu_{A})(B_{i}-\mu_{B}),$$

where *μ* is the mean value of each signal, and *N* is the number of samples. The correlation coefficient *ρ* is then calculated by normalization, using the standard deviation of both signals,

5.2 $$\rho =\frac{\mathrm{cov}(A,B)}{\sigma_{A}\sigma_{B}}.$$

**Figure 8.**
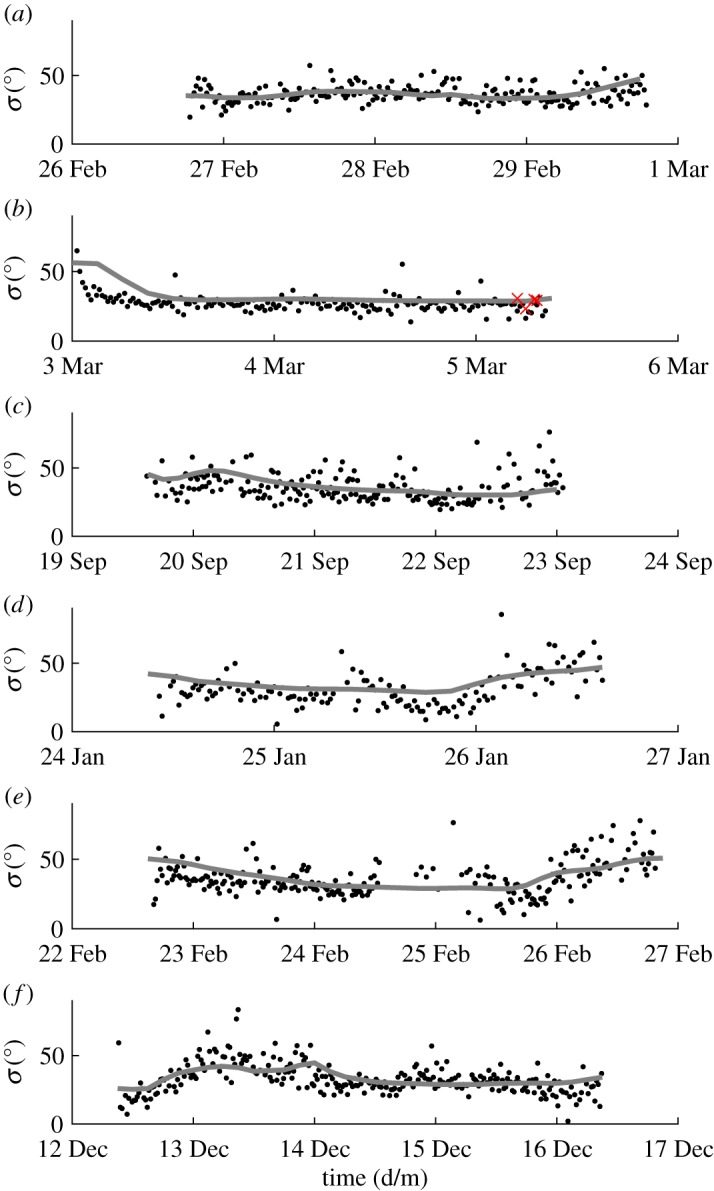
LWM spreading estimate and ECMWF hindcast predictions with time. (*a*) Storm 28, (*b*) Storm 30, (*c*) Storm 134, (*d*) Storm 301, (*e*) Storm 320 and (*f*) Storm 435 as detailed in table 3: *σ*_opt_ (black dots); *σ*_E_ (grey line); and where available *σ*_*D*_ (×). (Online version in colour.)

Figure 8 shows the temporal behaviour of the ‘long wave estimate’ of spreading obtained for six storms chosen from the dataset. These storms were selected because they exhibited the strongest correlation between *σ*_opt_ and *σ*_E_ corresponding to the largest values of *ρ*, while having significant temporal variation over their duration. Effects of random uncertainty and/or noise are evident in the values of *σ*_opt_. This variability stems from noise that is naturally found in real measurements, causing the standard deviation of the resulting estimates to increase. Adcock & Taylor [14] used numerically generated examples with artificially added noise to demonstrate that the standard deviation of the resulting values of *σ*_opt_ increased as the signal-to-noise ratio decreased. However, the mean value of the estimates remained correct.

Noise contained within the values of *σ*_opt_ has an effect on the calculation of *ρ*. For two perfectly correlated signals where *ρ* is 1, the introduction of random errors invariably reduces the value of *ρ* [30]. To achieve a better understanding of the true correlation of the results, raw values of *σ*_opt_ were smoothed in the time domain with local regression using weighted linear least squares and a second degree polynomial model. Table 3 lists values of *ρ* for both the smoothed and raw LWM estimates. For the raw results, the values of *ρ* are relatively low due to noise in the data. Even so, ρ⪆0.5, except for storms 28 and 134, meaning a weak correlation exists. After data smoothing, all the values of *ρ* are well above 0.5, with some approaching unity, illustrating a strong correlation between *σ*_opt_ and *σ*_E_.

**Table 3.** Fit parameters of well-correlated storms, correlation coefficient *ρ* for raw and smoothed estimates, and number of high-quality time series *N*_*QC*_.

			*ρ*		
storm	start	end	raw	smoothed	*N*_*QC*_
28	9 Feb 2000 08.58.28	14 Feb 2000 02.39.12	0.268	0.653	207
30	3 Mar 2000 02.14.06	5 Mar 2000 02.34.26	0.528	0.917	166
134	19 Sep 2000 14.47.46	22 Sep 2000 04.32.10	0.316	0.775	196
301	24 Jan 2002 10.21.18	26 Jan 2002 03.21.28	0.648	0.889	140
320	22 Feb 2002 16.09.18	26 Feb 2002 03.29.50	0.494	0.809	222
435	12 Dec 2003 09.12.14	16 Dec 2003 03.32.46	0.566	0.759	265

Not all of the storms in the dataset show such strong correlation. The indirect nature of the hindcast data means that negative comparisons are fairly inconclusive. However, when the hindcast and measurements exhibit high correlation, this provides conclusive evidence that the LWM is capturing the dynamically changing directional conditions being modelled by the hindcast. It is very unlikely that these two predictions correlate by pure coincidence without being an accurate measure of actual observed conditions.

### (b) Comparison with iterative maximum-likelihood method estimates

**Figure 9.**
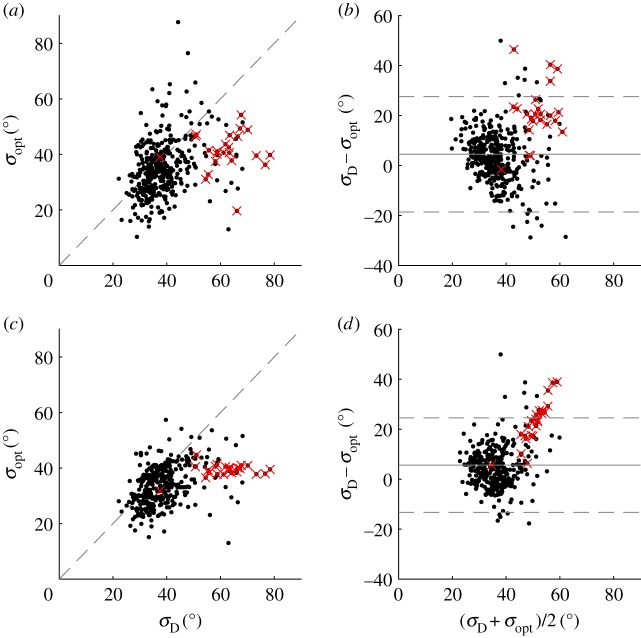
Comparison of IMLM and LWM estimates: scatter plot of *σ*_opt_ against *σ*_D_, (*a*) raw *σ*_opt_, and (*c*) smoothed *σ*_opt_; and Bland–Altman plot of mean spreading against difference in spreading estimates, (*b*) raw *σ*_opt_, and (*d*) smoothed *σ*_opt_. Superimposed mean difference (grey solid line), two standard deviation limits of agreement (grey dashed line), all 386 analysed data (black dots), and the results from storm 18 (crosses). (Online version in colour.)

Of the 448 storms considered herein, 66 storms contain a total of 391 individual measurements for which all three probes pass the quality control process, allowing estimation of *σ*_D_. This represents a very small portion of the entire dataset of 16 422 measurements. Unlike the hindcast data estimate *σ*_E_, calculation of *σ*_D_ provides a direct measurement of local spreading. Figure 9*a* shows a direct comparison between the values of *σ*_D_ and *σ*_opt_ calculated for each set of concurrent measurements. The data are scattered about the line *σ*_D_=*σ*_opt_; as the apparent value of spreading increases, the agreement between the two estimates appears to reduce. Figure 9*b* presents the Bland–Altman plot which displays the difference between *σ*_D_ and *σ*_opt_ against their average value. The Bland–Altman plot illustrates the uncertainty inherent to both sets of estimates and the relationship between magnitude and level of agreement, without assuming that one method is better than the other [31]. The mean difference for all data, shown by the solid grey line, is 4.98°; this represents the bias between the two methods. As the mean value increases, the agreement between the two methods reduces, and the uncertainty increases. The dashed lines show 95% confidence intervals, which are calculated as twice the standard deviation from the mean; the majority of the data sit within these limits.

Given that the LWM requires only one measurement for each estimate, the number of available estimates means that it is possible to reduce the effects of random uncertainty by smoothing the data in the time domain. Smoothing is achieved using the same approach described in §5a. Figure 9*c*,*d* shows the effect of smoothing the LWM results on the agreement between the two methods. This removes some of the uncertainty associated with *σ*_opt_ (as addressed in the previous section) and has two effects. Firstly, the agreement increases for the majority of the data, as evident in figure 9*d* where the data are more tightly clustered about the mean. Secondly, it serves to highlight the uncertainty associated with the values of *σ*_D_. As the mean value of spreading increases, agreement decreases with a positive bias, owing to the larger values of *σ*_D_.

**Figure 10.**
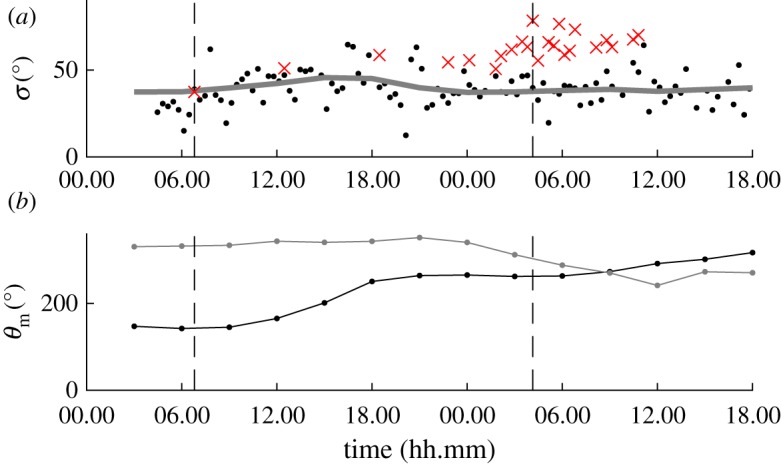
Wave spreading results for Storm 18 which exhibit significant disagreement between *σ*_opt_ and *σ*_D_: (*a*) *σ*_opt_ (black dots), *σ*_E_ (grey line) and *σ*_D_ (×); and (*b*) mean directions of wind waves (black) and total swell (grey). Dashed lines in both plots indicate the measurement times of figures 11*a*,*b*. (Online version in colour.)

**Figure 11.**
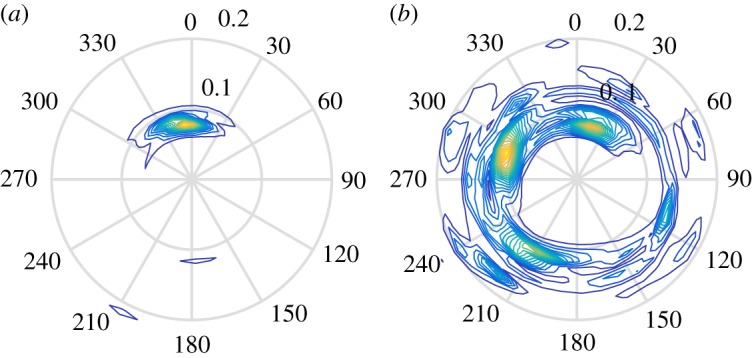
Frequency-dependent direction spectrum measured during Storm 18, calculated using IMLM: (*a*) 06.47 1 February 2000; (*b*) 04.08 2 February 2000. (Online version in colour.)

Considering the relatively small sample size, the comparison is sensitive to the presence of outliers in the data. The majority of outliers stem from Storm 18. Figure 10*a* presents IMLM, LWM and ECMWF hindcast estimates of spreading over the duration of the storm. In this storm, both *σ*_opt_ and *σ*_E_ follow the same general trend, whereas in the latter half of the storm *σ*_D_ presents larger values (about 20° greater). The correlation between *σ*_opt_ and *σ*_E_ may suggest that the IMLM is in error in this case. The apparent error in *σ*_D_ may be a result of crossing-wave conditions. Figure 10*b* shows the mean directions of wind and swell waves predicted by the ECMWF hindcast for Storm 18. The hindcast results suggest that the predominant wind and swell waves were propagating in quite different directions during the storm. Therefore, the complex crossing conditions are being detected by the frequency-dependent IMLM causing an increase in the value *σ*_D_. The simplicity of the assumed frequency-dependent spreading distribution used by LWM only allows for the detection of the average spreading about the mean direction. This is highlighted by figure 11, which shows frequency-dependent directional spectra produced by the IMLM at the start and one day into the storm, indicated by dashed vertical lines in figure 10. Figure 11*a* shows the spectrum calculated from measurements made at 06.47 on 1 February 2000. At this point in the storm, the spectrum has an obvious predominant direction with no signs of major crossing components, and the spreading estimates made by all three sources agree well, as illustrated in figure 10*a*. Figure 11*b* depicts the spectrum calculated at 04.08 on 2 February 2000. The corresponding spreading estimates shown in figure 10*a* differ significantly (*σ*_D_=78.5°, *σ*_E_=37.8° and *σ*_opt_=39.8°), and the directional spectrum exhibits several crossing wind and swell components.

**Figure 12.**
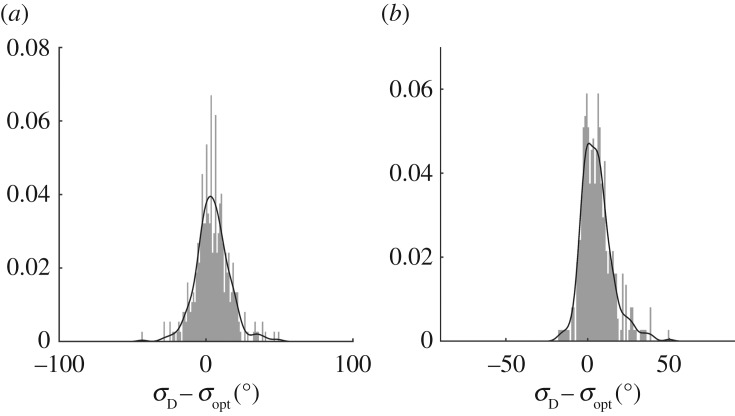
Histograms and kernel density of difference in wave spreading estimated by LWM and IMLM methods: (*a*) raw *σ*_opt_; and (*b*) smoothed *σ*_opt_.

Figure 9 shows that the results from IMLM and LWM have mean bias of 5.0°, and are in close agreement which reduces slightly as spreading increases. The scatter of results indicates there is uncertainty associated with both methods. Figure 12 presents the histograms and kernel density estimates of error between IMLM estimates and both raw, and smoothed LWM results. The histogram in figure 12*a*, for the raw LWM results, displays symmetry with a normal distribution, suggesting the errors primarily arise from random noise. The histogram in figure 12*b* is slightly asymmetric as a result of smoothing *σ*_opt_, and exhibits better agreement as a narrowing of the distribution.

**Figure 13.**
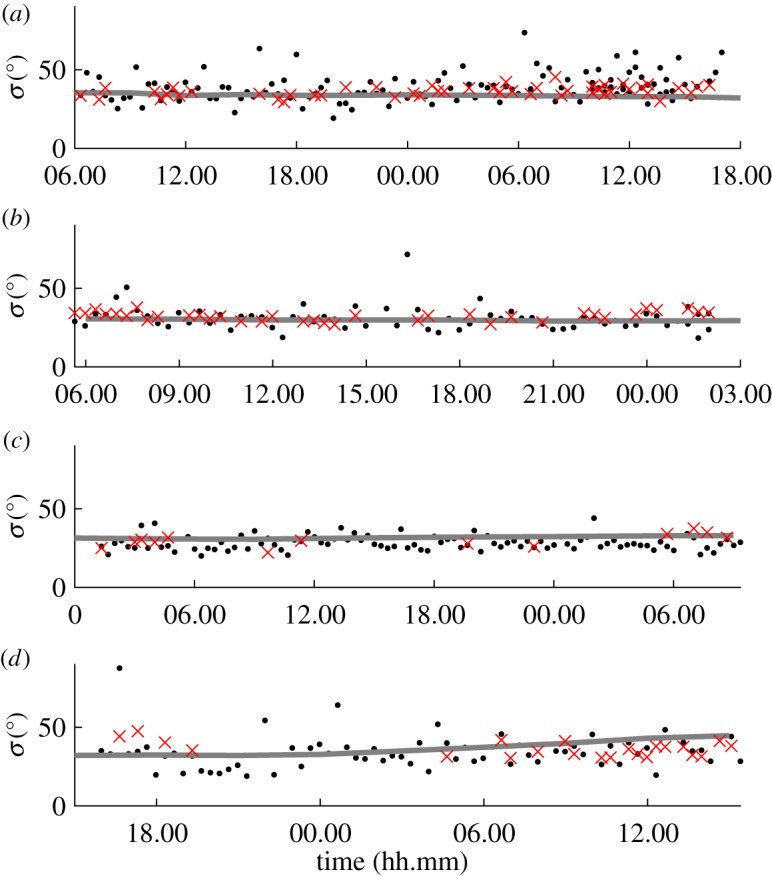
LWM, IMLM, and ECMWF hindcast predictions of wave spreading for (*a*) Storm 3, (*b*) Storm 57, (*c*) Storm 369 and (*d*) Storm 448 as detailed in table 4: *σ*_opt_ (black dots), *σ*_D_ (×) and *σ*_E_ (grey line). (Online version in colour.)

In the dataset, there are four storms for which IMLM estimation is possible over their duration and a better understanding can thus be gained of the relative uncertainty of the LWM and IMLM methods. Figure 13 displays the four storms detailed in table 4. The first section of Storm 3 was included, and the remainder rejected, in order to preserve data quality and the relative stationarity of spreading. For all four storms, *σ*_opt_ (black) and *σ*_D_ (×) follow the same trend as the hindcast predictions *σ*_E_, with relatively little temporal variation. These examples further reinforce that LWM has correctly estimated the local spreading given that all three estimates are in good agreement. In figure 13*d*, there is a slight time lag between the values of *σ*_E_ and the other two estimates which are better correlated. This storm also shows slightly more variation in spreading, whereas the other storms remain virtually stationary. Table 5 lists values of the mean and standard deviation of the spreading estimates obtained using IMLM and LWM for the four storms. Where the value of spreading remains constant, the standard deviation of each set of estimates provides a satisfactory analogue to uncertainty associated with the particular method employed, assuming that a near-constant value of spreading is a true representation of the actual conditions. Dividing the standard deviation by the square root of the number of samples gives the standard error (s.e.), which is a measure of variability accounting for the number of samples [32]. The standard error of *σ*_opt_ is roughly two to three times that of *σ*_D_ for all but Storm 369 where *σ*_opt_ is just over half the value of *σ*_D_. This storm happens to have the lowest spreading value predicted by both methods (*σ*_opt_=27.9°, *σ*_D_=30.5° and *σ*_E_=31.8°).

**Table 4.** Details of selected storms with IMLM directional data, showing the number of high-quality time series *N*_*QC*_ and directional data *N*_*Dir*_.

storm	start	end	*N*_*QC*_	*N*_*Dir*_
3*	23 Mar 1997 06.19.14	29 Mar 1997 09.26.32	851	110
57	1 Apr 2000 05.38.48	2 Apr 2000 01.58.58	161	36
369	22 Nov 2002 03.40.26	23 Nov 2002 03.20.38	202	15
448	26 Sep 1996 15.58.30	27 Sep 1996 06.38.30	148	22

**Table 5.** Fit parameters of IMLM storms, standard deviation (s.d.), standard error (s.e.), and mean *σ*_*m*_ for all three estimates of spreading.

	s.d.			s.e.			*σ*_*m*_		
storm	*σ*_opt_	*σ*_D_	*σ*_E_	*σ*_opt_	*σ*_D_	*σ*_E_	*σ*_opt_	*σ*_D_	*σ*_E_
3	8.87	3.29	0.919	0.820	0.208	0.255	38.1	36.3	33.7
57	7.90	2.94	0.496	0.987	0.298	0.175	30.6	32.2	29.8
369	4.39	3.93	0.819	0.450	0.744	0.237	27.9	30.5	31.8
448	9.93	4.61	4.92	1.20	0.610	1.64	33.5	35.7	36.6

**Figure 14.**
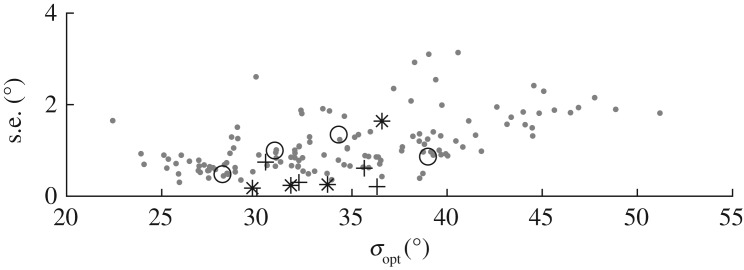
Uncertainty measured by standard error versus mean predicted value of spreading for each storm: mean *σ*_opt_ (grey dots); storms from table 4 *σ*_opt_ (°), *σ*_D_ (+) and *σ*_E_ (×).

Making the assumption that the smoothed value of *σ*_opt_ for each storm is the actual local spreading, the data can be de-trended and a similar analysis performed on the entire dataset. Figure 14 shows how the standard error of *σ*_opt_ varies with the mean value of *σ*_opt_ over the duration of each storm plotted as grey dots. The values for the four storms plotted in figure 13 and listed in table 4 are overlaid. There is a clear positive correlation between the predicted spreading and the standard error. This is an intuitive result because the simplified assumption of frequency-independent wrapped-normal spreading becomes less valid as spreading increases.

As discussed in §5a, there are a number of estimates *σ*_opt_<20° which are not within the range of *σ*_E_. Figure 9*a* also shows values of *σ*_opt_<20°; however, after smoothing the number reduces (figure 9*c*). Figure 14 shows that no storms with multiple concurrent measurements have a mean value of *σ*_opt_<20°, therefore these values are most likely the result of noise.

## 6. Conclusion

Adcock & Taylor [14] devised a means of determining directional spreading, the LWM, which they verified for several deterministic cases and later applied to the 1 January 1995 Draupner wave [16]. Herein, we extend the approach of the LWM to a large dataset of *in situ* ocean observations of free surface elevation obtained from the North Alwyn platform in the northern North Sea. The approach taken to implement the LWM, greatly reduced its computational cost. The LWM analysis was based on an assumption that directional spreading was independent of frequency, and excluded records containing freak waves. A method for detecting the influence of erroneous measurements is also presented, which allows *a posteriori* identification of spurious values of estimated spreading. The results of the LWM are compared with values predicted by the ECMWF hindcast model using the North Alwyn data. Despite the indirect nature of this comparison, good temporal correlation is found between the two estimates of spreading for selected storms. This provides confidence in the ability of the LWM to track dynamically varying directional spreading conditions. By comparing results using a small sample of the overall dataset, it is found that close agreement is achieved between the spreading estimates made by LWM and the IMLM, with the former giving smaller values indicating a slight bias. The LWM exhibits slightly greater uncertainty than the IMLM; however, given that LWM requires only a single measurement, the uncertainty can be reduced by averaging. To gain better understanding of the small bias that exists between the two measurement approaches and the performance of LWM at values of *σ*<20°, it is recommended to extend the analysis in future to a dataset containing a much higher proportion of accurate, concurrent measurements. This would enable a quantitative assessment of the relative uncertainty of both methods. This study has demonstrated that the LWM can be effectively extended to noisy real sea observations, such as prevail at North Alwyn. Available additional information has been used to provide confidence in the accuracy of the results. As with the conventional IMLM method, the LWM is susceptible to the effects of noise which manifests itself in the form of random uncertainty. However, where sufficient results exist, averaging effectively removes uncertainty without requirement of further post-processing. In short, the study has provided further confirmation that the LWM is a viable alternative to conventional means of directional spreading estimation. In practice, LWM offers a very promising opportunity to gain vital directional information on ocean waves from single-point measurements.

## Data accessibility

The datasets used herein are owned and distributed by Total S.A, and the ECMWF.
